# Food Insecurity Among Older Adults: A Transprofessional, Community-Based Collaboration

**DOI:** 10.1093/geroni/igaa057.122

**Published:** 2020-12-16

**Authors:** Shirley Girouard, Michele Solloway

**Affiliations:** SUNY Downstate Health Sciences University, Brooklyn, New York, United States

## Abstract

To address inequities related to food insecurity among older adults, a better understanding of the phenomenon was needed. An innovative screening tool to distinguish among high, medium and low risk and that considers cultural preferences related to food acquisition and related behaviors was developed and piloted. Screenings and healthy eating education were offered at health fairs and other community events. Information about food insecurity and healthy eating as well as resources, such as food maps, guidelines, and food preparation materials were disseminated. Preliminary findings suggest that over half of those screened have high levels of food insecurity. Approximately 37% have five or more comorbidities that combined with food insecurity, represent a significant threat to health and well-being. Elected officials and community leaders soon learned about this initiative and sought education and screening for their constituencies. The research and project evaluation will be used in collaboration with these leaders to identify polices at the local, state and federal level to promote health equity and reduce food insecurity disparities. Efforts are under development to integrate the new screening and referral mechanisms in community-based primary care practices.

